# Viperin and Its Effect on SVCV Replication in Common Carp, *Cyprinus carpio*

**DOI:** 10.3390/ani15010096

**Published:** 2025-01-03

**Authors:** Yan Meng, Xi Hu, Nan Jiang, Yuding Fan, Yiqun Li, Mingyang Xue, Chen Xu, Wenzhi Liu, Yong Zhou

**Affiliations:** Yangtze River Fisheries Research Institute, Chinese Academy of Fishery Sciences, Wuhan 430223, China; mengy@yfi.ac.cn (Y.M.); hxzcto@163.com (X.H.); jn851027@yfi.ac.cn (N.J.); fanyd@yfi.ac.cn (Y.F.); liyq@yfi.ac.cn (Y.L.); xmy@yfi.ac.cn (M.X.); xuchen@yfi.ac.cn (C.X.); liuwenzhialisa@yfi.ac.cn (W.L.)

**Keywords:** common carp (*Cyprinus carpio*), *viperin*, characteristics, antiviral activities

## Abstract

*Viperin* is an interferon-stimulated gene (ISG) that plays an important role in the congenital antiviral immunity of vertebrates. In this study, the common carp *viperin* (*cc-viperin*) gene is characterized, and we determine whether it has the ability to inhibit spring viremia of carp virus (SVCV) replication in EPC cells. The results showed that the full-length cDNA of the *cc-viperin* gene was 1044 bp and it encoded 348 amino acids. The cc-viperin sequence contained a leucine zipper in the N-terminal, a CxxxCxxC motif in the SAM domain, and a conservative C-terminus. The *cc-viperin* gene’s nucleotide and amino acid sequence alignment revealed that *cc-viperin* displayed relatively high sequence identity compared with other species. Phylogenetic analysis displayed the close relation of cc-viperin with *Carassius auratus* and *Mylopharyngodon piceus*. Subcellular localization analysis indicated that the cc-viperin protein was located in the cytoplasm. The gene expression results showed that *cc-viperin* was expressed in all of the tissues tested. Its expression level significantly increased in EPC cells after 24 h to 72 h compared to the control during SVCV infection. Moreover, *cc-viperin* significantly inhibited SVCV replication when it was overexpressed, whereas it increased SVCV replication when it had reduced expression in EPC cells, respectively. To summarize, the results obtained in this work show that *cc-viperin* shares similar sequence characteristics with other vertebrates, and it could inhibit SVCV replication in EPC cells, displaying an antiviral effect in common carp.

## Simple Summary

*Viperin*, as an important ISG, has important roles in antiviral immunity. In this paper, the common carp *viperin* (*cc-viperin*) gene is characterized and analyzed. The *cc-viperin* gene’s nucleotide and amino acid sequence alignment reveals that cc-viperin displays relatively high sequence identity compared with other species. It is located in cytoplasm. It is expressed in most tissues. It could inhibit spring viremia of carp virus (SVCV) replication in EPC cells, showing an antiviral effect of viprein. This research is not only an extension of *viperin* gene research but also contributes to basic research on viral diseases in aquatic animals.

## 1. Introduction

The first action of cells in responding to viral infections is activating the innate immune pathways. The interferon (IFN) system is the core of the innate immune system and has a broad cellular response to microbes. It can also be said that interferon is the first line of cells’ defense against pathogens. Interferon can induce hundreds of interferon-stimulated genes (ISGs) to interfere with viral replication when IFN is activated after a virus invades a cell. Many ISGs even possess direct antiviral properties [1]. Viperin is an important ISG product. It was first identified in 1997, during a human cytomegalovirus (HCMV) study, and therefore it was also originally called cytomegalovirus-inducible gene 5 (cig5) [2]. In addition, it was also known as radical SAM domain-containing 2 (RSAD2), because it was identical to the radical S-adenosyl methionine (SAM) domain-containing protein 2, which possesses the classical CxxxCxxC motif [3]. The now commonly used name, viperin, originated from its properties as a virus-inhibitory protein, which are endoplasmic reticulum-associated and interferon-inducible [4,5]. Viperin can interfere with the replication of diverse DNA and RNA viruses in organisms. Since it was first identified, viperin has been widely identified in the genomes of various mammals, birds, reptiles, and fish, which shows that it was highly conserved during evolution [6]. Generally, it is expressed at a low basal level in most cells; however, it can be significantly upregulated during viral infection or in IFN or LPS treatment [3,7,8]. In bony fish, *viperin* was first discovered by challenging hemorrhagic septicemia virus (VHSV) in rainbow trout (*Oncorhynchus mykiss*) [9]. Subsequently, it was found to be induced by viruses, bacteria, or other analogs, and it exists in various fish, such as *Carassius auratusgrass* [10], Atlantic cod [11], Atlantic salmon [12], tilapia [13], rock bream [14], red drum [15], crucian carp [5], zebrafish [16], large yellow croaker [17], and redlip mullet [18]. All of these studies showed viperin’s role in the resistance to pathogens or their analogs.

Common carp (*Cyprinus carpio*) is one of the most economically valuable freshwater fish species worldwide. The majority of the global production of common carp is destined for human consumption. Ornamental koi, a selective variety bred from common carp, is also a species with significant ornamental value in many countries [19]. According to statistics from the Food and Agriculture Organization (FAO) of the United Nations, common is carp ranked first, and its production amounted to 51.6% of the global major aquaculture species and major species of fish worldwide in 2022. China is the world leader in carp breeding. At present, at least 20 bred strains are cultivated in China [20]. The continued sustainability of the aquaculture industry depends on its profitability. Disease is an important factor affecting the yield, sustenance, and economic development of aquaculture [21]. Among the numerous diseases in common carp, the three viral diseases caused by carp edema virus (CEV), cyprinid herpesvirus 3 (koi herpesvirus, CyHV-3, KHV), and carp sprivivirus (spring viremia of carp virus, SVCV) seem to be the most important, as they have resulted in extensive damage and major losses in carp aquaculture [22]. Spring viremia of carp (SVC), with SVCV as its causative agent, is endemic in Europe, America, Asian countries, etc., where it causes significant morbidity and mortality in cyprinids, primarily in common carp [23]. SVCV infection is highly lethal in young fish, with mortality rates of up to 90%. The expansion of the infection area and host range of SVCV not only poses a threat to the fish trade industry but also to the safety of wild fish populations. Furthermore, unfortunately, there is neither a commercial vaccine for its control and prevention nor a specific medicine for its treatment. Research on the immunization and prevention of this viral disease is urgently needed [24].

In this study, we identified and characterized the full-length cDNA of the *viperin* gene in common carp (*cc-viperin*). Its subcellular localization and expression profiles in normal tissues and SVCV infection in EPC cells were also analyzed. The results indicated that the *cc-viperin* gene expression level was upregulated in EPC cells during SVCV infection. The gene and its protein, namely the SVCV glycoprotein, were significantly reduced in EPC cells with *cc-viperin* overexpression. However, when the *cc-viperin* expression in EPC cells was lowered by specific interfering RNA molecules, they were dramatically enhanced at different infection time points compared to the negative control cells. The results suggest that *cc-viperin* can respond to SVCV infection, exerting an antiviral effect during SVCV replication. This may provide new insights into the fish *viperin* regarding viral infection and provide a certain theoretical basis for virus prevention and control.

## 2. Materials and Methods

### 2.1. Animals, Cells, and Virus

Common carp (about 10 cm) was collected from the Yangtze River Fisheries Research Institute’s experimental farm (Hubei, China). The individuals were acclimatized in aerated fresh water tanks at 20 °C and fed daily for 2 weeks before the experiment. Six individuals were randomly selected to test the absence of SVCV. The *Epithelioma papulosum cyprini* (EPC) cell line and SVCV were preserved in our lab. SVCV was passaged in EPC cells incubated at 25 °C and fed with 5 mL M199 medium (Hyclone, Logan, UT, USA) containing 10% fetal bovine serum (FBS) (Every Green, Hangzhou, China) in a T-25 cm^2^ cell culture dish (Corning, NY, USA). EPC cells were infected with SVCV at a 0.01 multiplicity of infection (MOI) and absorbed for 1 h in 2% FBS M199 medium. Then, they were cultured in M199 medium with 5% FBS. SVCV replication was determined by a 50% tissue culture infective dose (TCID_50_/mL) assay using the Reed and Muench method [25]. All procedures complied with the rules of the Animal Care and Use Committee of the Institute (YFI2022MY002).

### 2.2. Viperin Cloning and Sequence Analysis

Total RNA was extracted from each tissue sample using an RNA extraction kit (Aidlab, Beijing, China). The RNA quality was evaluated by gel electrophoresis and spectrophotometry. The *viperin* gene of common carp was referenced to the genome sequence of common carp (*Cyprinus carpio*) [26]. Primers of Viperin-F/R for *viperin* gene amplification (shown in Table 1) were designed to verify it based on the conserved domain. The first-strand cDNA template was synthesized from the total RNA using a reverse transcription kit (TransGen, Beijing, China) and stored at −20 °C before usage. PCR amplification was performed according to the following procedure: denaturation at 95 °C for 10 min, followed by 35 cycles at 95 °C for 1 min, annealing at 55 °C for 1 min, and extension at 72 °C for 1 min; finally, further extension at 72 °C for 10 min was performed. The amplification product was detected, purified, and connected to a pMD18-T vector (TaKaRa, Dalian, China) for Sanger sequencing. The nucleotide and protein sequences were blasted in the NCBI database (https://blast.ncbi.nlm.nih.gov/Blast.cgi (accessed on 9 May 2023)). The *viperin* gene sequence was aligned using Clustal W in MEGA 7.0. The phylogenetic tree was constructed by MEGA 7.0 using the NJ method with 1000 bootstrap replicates. Its molecular weight (MW) and isoelectric point (pI) were predicted by EditSeq (DNASTAR, Inc. Madision, WI, USA). The subcellular localization and functional domain were predicted by Cell-PLoc 2.0 (http://www.csbio.sjtu.edu.cn/bioinf/Cell-PLoc-2/).

### 2.3. Viperin Gene Expression

The *viperin* gene expression profiles in normal tissue and in EPC cells infected with SVCV were determined by quantitative real-time PCR (RT-qPCR). The cells were harvested at 0, 6, 12, 24, 48, and 72 h after SVCV infection. The total RNA was isolated using a tissue/cell RNA rapid extraction kit from EPC cells or normal tissue from the heart, brain, gill, muscle, liver, kidney, and spleen. The relative expression of *viperin* was detected with the primers of Viperin-qF/R, and *β-actin* expression was detected with the primers of β-actinF/R as the internal control (Table 1). The RT-qPCR reaction was carried out using the SYBR^®^ Select Master Mix (2×) (TaKaRa, Dalian, China), according to the instructions, with the reaction system consisting of 10 µL mix, 2 µL cDNA, 1 µL of each primer, and 6 µL ultrapure water. The RT-qPCR reaction conditions were as follows: 10 min at 95 °C, 40 cycles of 95 °C for 15 s, 57 °C for 30 s, and 60 °C for 5 min on a Rotor-Gene 6000 Real-Time PCR system (Qiagen, Dusseldorf, Germany). The relative gene expression was calculated using the 2^−∆∆CT^ method.

### 2.4. Plasmid Construction and Transfection

The *viperin* gene sequence was amplified using the primer Viperin-F1/R1 (Table 1) and cloned into the vector pEGFP-N1 based on the specifications. The recombinant plasmid, named pEGFP-N1-Viperin, was cultured, and its DNA was extracted using the Endo-Free Plasmid Mini Kit (Omega, Norcross, GA, USA) to identify the sequence. EPC cells with a concentration of 5 × 10^6^ cells/mL were seeded into T-25 cm^2^ cell culture flasks and cultivated in M199 medium containing 10% FBS for 24 h. When a 70~90% cell monolayer was formed, the culture medium in each flask was replaced with 2 mL fresh M199 medium. pEGFP-N1 and pEGFP-N1-Viperin were used as the control group and experimental group. The transfection was carried out with 200 μL of M199 medium containing 2.5 μg plasmids/recombinant plasmids and 7.5 μL PolyJet™ (Signagen, Frederick, MD, USA) added into each flask, respectively. Cells treated only with PolyJet™ were used as blanks. After transfection for 6 h, the medium was replaced with fresh M199 medium containing 10% FBS for continuous cultivation. After 48 h, the transfection effects of pEGFP-N1-Viperin and pEGFP-N1 were observed with a fluorescent microscope. In addition, the protein expression of the recombinant plasmid pEGFP-N1-Viperin was detected by Western blotting using pEGFP-N1-Tag mouse polyclonal antibody (Abclonal, Guangzhou, China). Meanwhile, the β-actin protein expression was detected using anti-β-actin mouse polyclonal antibody as the control reference (CST, Boston, MA, USA). Briefly, the cells were harvested and lysed with SDS Lysis Buffer (Beyotime, Shanghai, China), and the suspension was mixed with sodium dodecyl sulfate–polyacrylamide gel electrophoresis (SDS-PAGE) loading buffer (volume 1:1) under heating at 95 °C for 10 min. Then, the protein samples were separated by 12% SDS-PAGE for 2 h and then electrotransferred to polyvinylidene fluoride (PVDF) membranes using a semidry blotter (Bio-Rad, Hercules, CA, USA). Subsequently, the membrane was blocked with 5% skim milk TBST (0.1% Tween-20 in Tris-buffered saline, pH 7.5) at room temperature for 2 h. It was then incubated with antibodies at a dilution of 1:5000 overnight at 4 °C. After being washed with TBST three times, the membrane was incubated with a secondary antibody and washed again with TBST three times. Finally, the membrane was placed in the Western Lightning ECL substrate system (Perkin Elmer, Waltham, MA, USA) before exposure with an imaging system (Bio-Rad, Hercules, CA, USA).

### 2.5. Viperin Subcellular Localization

pEGFP-N1-Viperin and pEGFP-N1 were set as the experimental group and control group. The transfer process was as described above. Cells were seeded in glass-bottom dishes (Biosharp, Hefei, China). After transfection for 48 h, the cell culture medium was removed. The EPC cells were washed with PBS (Hyclone, Logan, UT, USA) three times and fixed in 4% paraformaldehyde for 20 min. Then, they were stained with 6-diamidino-2-phenylindole (DAPI) (Solarbio, Beijing, China). Lastly, the cells in the dishes were washed with PBS and detected with a confocal microscope (Olympus, Tokyo, Japan).

### 2.6. Effect of Viperin Overexpression on SVCV Replication

EPC cells were cultured in 6-well plates for 18–24 h and divided into an experimental and control group. After transfection for 48 h, the cells were infected with SVCV and then virus inoculation was performed. The cells were collected at 24 h, 48 h, and 72 h after inoculation. The total RNA was extracted and reversed into cDNA, and the mRNA and protein expression levels of SVCV-G at each time period were detected by RT-qPCR and Western blot, respectively. The SVCV RT-qPCR primer, reaction system, and amplification procedure referred to the study of Wang et al. [27]. The total RNA was isolated using a tissue/cell RNA rapid extraction kit and further RT-qPCR analysis. RT-qPCR was carried out to detect the G protein of SVCV using the primer qSVCV-F/qSVCV-R (Table 1) in the Rotor-Gene 6000 Real-Time PCR system. The gene expression was calculated by the 2^−ΔΔCT^ method, and *β-actin* was used as an internal control [28]. The protein samples were processed as follows. First, 100 μL lysate was added to the cells to lyse them. Then, 25 μL of 5× SDS loading buffer was added, and the sample was mixed well and boiled for 10 min. The samples were separated by 10% SDS-PAGE and transferred onto 0.45 μm pore nitrocellulose membranes via a semi-dry blotter (Bio-Rad, Hercules, CA, USA). The membranes were blocked in freshly prepared TBSA (2.5% BSA in TBST buffer) for 2 h and, respectively, incubated with the anti-SVCV-G mouse monoclonal antibody (1:5000) and the anti-β-actin rabbit monoclonal antibody (ABclonal, Guangzhou, China) at a 1:5000 dilution for the loading control at 4 °C overnight. Then, the membrane was washed three times with TBST. The membrane containing SVCV-G mouse monoclonal antibody and β-actin rabbit monoclonal antibody was incubated with alkaline horseradish peroxidase-conjugated anti-mouse IgG and alkaline horseradish peroxidase-conjugated anti-rabbit IgG (CST, Boston, MA, USA), respectively, for 2 h at room temperature; it was then washed three times using TBST. Finally, the samples were incubated for 5 min in the Clarity™ Western ECL substrate (Bio-Rad, Hercules, CA, USA) and photographed by a gel imaging system (Bio-Rad, Hercules, CA, USA). A protein marker of 25–90 kDa (TransGen, Beijing, China) was used as a reference in the experiment. 

### 2.7. Synthesis, RNA Interference, and Effect Evaluation of shRNA

Targeting the carp *viperin* gene, three specific interfering RNA molecules (shViperins) and one unrelated interfering RNA molecule (shNC) were designed and synthesized (Suzhou Jima Company, Suzhou, China). The sequences are listed in Table 1. Three synthetic shRNA plasmids (shRNA−179, shRNA−756, and shRNA−1016) and a control plasmid (shRNA-NC) were transfected into the EPC cells. The fluorescence efficiency was observed by an inverted fluorescence microscope after transfection for 48 h. Western blot was used to detect whether the three shRNA plasmids were successfully transfected into the EPC cells. RT-qPCR was used to determine the effect of interference with viperin in order to screen the shRNA sequences with the best interfering effects.

### 2.8. Effect of Downregulation of Viperin on SVCV Replication

Effective interference sequences were transfected into EPC cells. After transfection for 48 h, SVCV was injected. The control group was given the same amount of M199 medium. Three samples were collected at 24, 48, and 72 h after SVCV infection. The *viperin* mRNA and protein expression profiles were detected as described above. 

### 2.9. Statistical Analysis

All data were subjected to statistical analyses by the GraphPad Prism 6.01 software. One-way analysis of variance (ANOVA) was employed for different group comparisons. The deviation was expressed as the mean ± SD (standard deviation). A *p*-value < 0.05 was considered statistically significant, and a *p*-value < 0.01 was considered extremely statistically significant. The ImageJ software (ImageJ 1.53c) was used for the grayscale analysis of the Western blot.

## 3. Results

### 3.1. Sequence and Analysis

**Figure 1:**
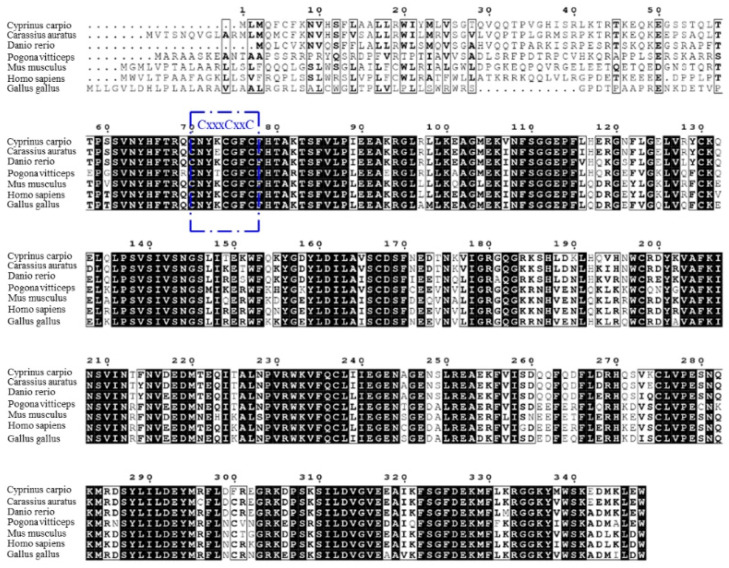
Multiple sequence alignment among the *viperin* gene-encoding proteins was performed with the Clustal W program. The three-cysteine motif CxxxCxxC is framed in blue. Consensus residues that are ≥75% identical among the aligned sequences are shaded in black.

According to the results, the full-length cDNA of common carp viperin was 1044 bp long and encoded a 348-amino-acid protein. The *viperin* gene of common carp was named *cc-viperin*. Its calculated molecular weight was 40.4 kDa and the theoretical isoelectric point was 8.027. Its amino acid alignment showed that the *cc-viperin* gene sequence contained a radical SAM domain (from 60 to 268 amino acids) with a three-cysteine motif CxxxCxxC. Compared the cDNA sequences of the crucian carp (*Carassius auratus*), rhinoceros (*Pogona vitticeps*), human (*Homo sapiens*), zebrafish (*Danio rerio*), Gallus domesticus (*Gallus gallus*), and bearded dragon (*Pogona vitticeps*), the sequence homology was 64.9% to 90.8%, and there were large differences in 70 amino acid regions of the N-terminal among them (Figure 1).

**Figure 2:**
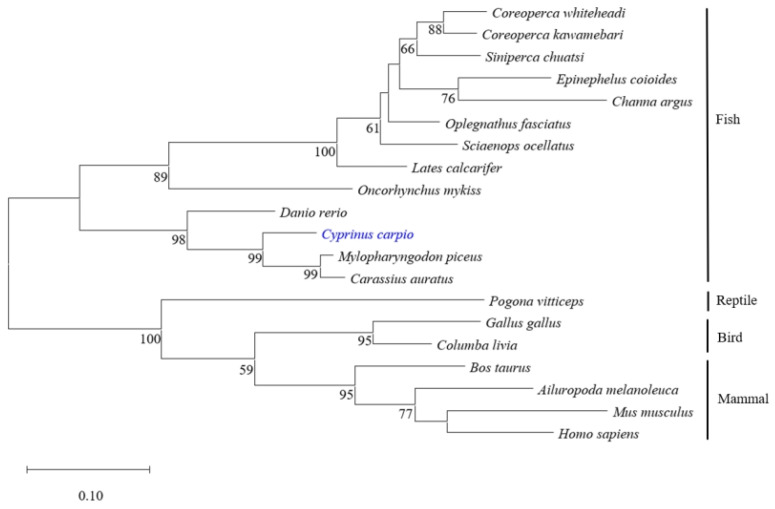
Phylogenetic analysis of different viperin protein sequences. The phylogenetic tree was constructed using the neighbor-joining method. Numbers beside the internal branches indicate bootstrap vales of 1000 replications. cc-viperin is marked in blue.

Different protein sequences of viperin were selected from the GenBank database to construct the phylogenetic tree by the NJ method. The results showed that all viperin sequences from fish were clustered in one group and separated from other species’ groups. The common carp viperin was closely related to that of crucian carp and black carp (*Mylopharyngodon piceus*) (Figure 2).

### 3.2. Viperin Expression Profiles in Tissue

**Figure 3:**
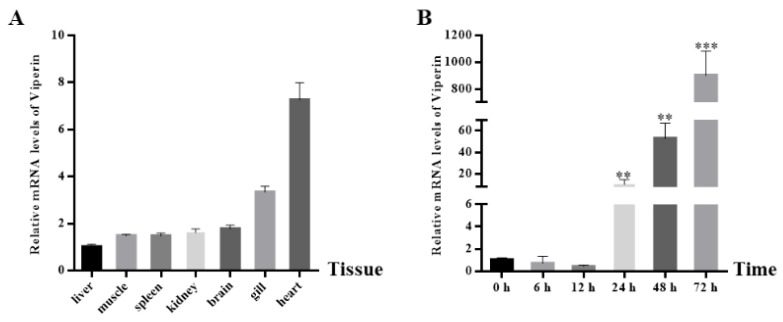
The *cc-viperin* gene expression in normal tissue and EPC cells during different SVCV infection periods. (**A**) The *cc-viperin* expression in healthy tissue, as analyzed by RT-qPCR. (**B**) The *cc-viperin* expression in EPC cells during different time periods of SVCV infection. Differences between the treated and control groups are marked with ** (*p* < 0.01) and *** (*p* < 0.001) to indicate significance.

The *cc-viperin* gene expression in the tissues of healthy common carp was determined by RT-qPCR (Figure 3A). The results showed that *cc-viperin* existed in all tissues tested. The highest expression level was found in the heart, while the lowest expression level occurred in the liver. The expression levels in the kidney and brain were similar. Compared to the control group, when EPC cells were infected with SVCV, the *viperin* gene expression level at different infection times showed an upward tendency. It showed less expression at 6 h and 12 h after infection and significantly increased at 24 h. The *viperin* expression level increased continuously until it reached the peak level at 72 h (Figure 3B).

### 3.3. Subcellular Localization of Viperin

**Figure 4:**
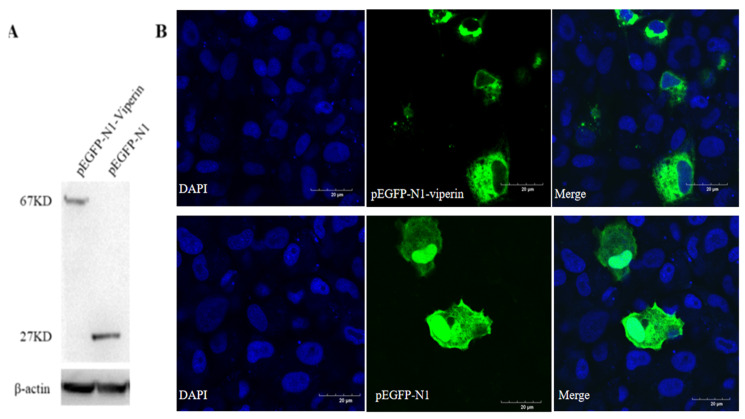
Viperin transfected in EPC cells detected by WB and subcellular localization observed by confocal microscopy. (**A**) EPC cells were transfected with pEGFP-N1-Viperin plasmid for 48 h, with pEGFP-N1 as a negative control. Mouse anti-GFP-Tag and anti-β-actin monoclonal antibody was used as the primary antibody, respectively. HRP-tagged anti-mouse IgG was used as the secondary antibody. (**B**) Subcellular localization of pEGFP-N1-Viperin in EPC cells stained by DAPI at 48 h post-transfection and observed by confocal microscopy. Scale bar: 20 μm. The original Western Blot images can be found in the Supplementary File.

The recombinant viperin protein in EPC cells transfected with pEGFP-N1-Viperin showed a molecular weight of 67 kDa, while the one transfected with the pEGFP-N1 plasmid displayed a band with a molecular weight of approximately 27 kDa (Figure 4A). Confocal microscopy observations showed that the viperin protein was mainly located in the cytoplasm (Figure 4B). It was a cytoplasmic protein.

### 3.4. Viperin Overexpression and SVCV Replication

**Figure 5:**
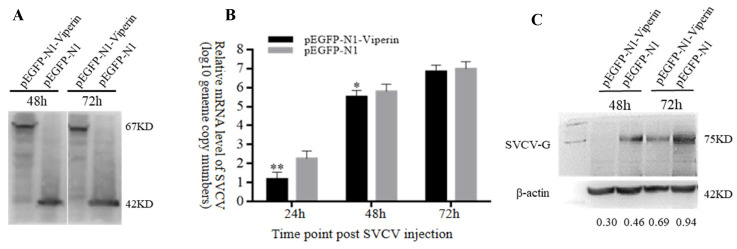
*Viperin* overexpression inhibited SVCV replication in EPC cells. (**A**) Stable levels of pEGFP-N1-Viperin fusion protein at different time points after SVCV infection. (**B**) The relative expression levels of SVCV-G determined by RT-qPCR. (**C**) The SVCV-G protein at different time points after SVCV injection by Western blot analysis. Differences between the treated and control groups are marked with * (*p* < 0.05) and ** (*p* < 0.01) to indicate significance. The original Western Blot images can be found in the Supplementary File.

In order to analyze the effect of *viperin* on SVCV replication, the mRNA and protein expression levels of SVCV-G were detected by RT-qPCR and Western blot, respectively, based on the test of viperin’s overexpression in EPC cells. The Western blot results indicated that pEGFP-N1-Viperin and pEGFP-N1 were stably expressed during SVCV infection (Figure 5A). Compared to the control group, the gene expression results demonstrated that the SVCV-G gene’s mRNA expression significantly decreased at 24 h and 48 h after SVCV infection (Figure 5B). In addition, compared to the control group, the Western blot showed that the expression of the SVCV-G protein decreased significantly at 48 h and 72 h after SVCV infection (Figure 5C). These results imply that the overexpression of *viperin* can inhibit SVCV replication in EPC cells.

### 3.5. Effect of shRNA Interference on Viperin Expression

**Figure 6:**
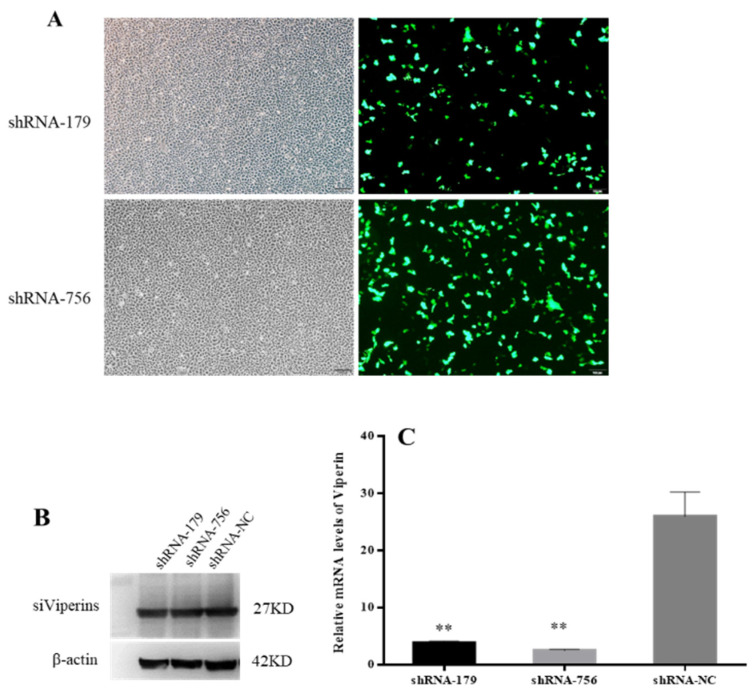
Effect of shRNA interference on *viperin* expression was detected by microscopy, Western blot, and qRT-PCR. (**A**) EPC cells were transfected with viperin-interfering molecules (shViperins) and unrelated interfering molecules (shNC) for 48 h, and the transfection efficiency was detected by inverted fluorescence microscopy. (**B**) Transfection of shViperins was detected by Western blot. (**C**) The viperin transcript levels detected by RT-qPCR after transfection. Differences between the treated and control groups are marked with ** (*p* < 0.01) to indicate significance. Scale bar, 100 μm. The original Western Blot images can be found in the Supplementary File.

The inverted fluorescence microscopy results showed that shRNA−179 and shRNA−756 had more green fluorescence (Figure 6A), while shRNA-1016 had low fluorescence efficiency, so the shRNA−179 and shRNA−756 plasmids were selected for subsequent experiments. The Western bolt results showed that shRNA−179, shRNA−756, and the control group had an obvious band at 27 KD. β-Actin showed an obvious band at 42 KD in both the experimental group and control group (Figure 6B). These results indicate that shRNA−179, shRNA−756, and shRNA-NC were successfully transfected into EPC cells. Moreover, the RT-qPCR showed that the *viperin* gene expression levels in the shViperin groups were obviously downregulated after transfection for 48 h (Figure 6C). Therefore, it can be concluded that the shRNA−179 and shRNA−756 plasmids, when transfected into EPC cells, can reduce the *cc-viperin* gene expression in these cells.

### 3.6. Effect of Viperin Downregulation on SVCV Replication

**Figure 7:**
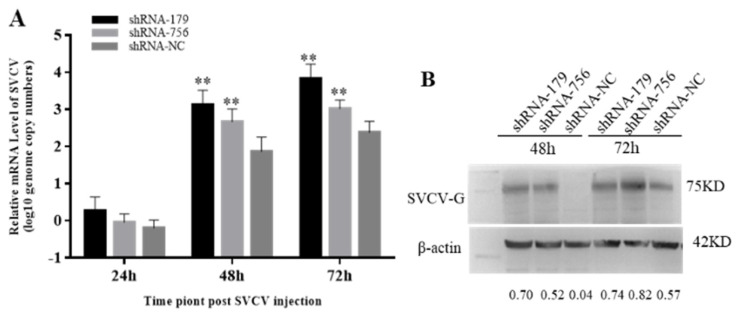
Downregulation of viperin by shRNA−179 and shRNA−756 promoted SVCV replication in EPC cells. (**A**) The relative mRNA level of SVCV was determined by RT-qPCR. (**B**) The SVCV-G protein at different time points after SVCV injection by Western blot analysis. Differences are marked with ** (*p* < 0.01) to indicate significance. The original Western Blot images can be found in the Supplementary File.

Since *viperin* overexpression can enhance SVCV replication, it was speculated that the endogenous viperin would have antiviral activity. When the *viperin* expression is downregulated, it could lead to a weak antiviral response in host cells. Therefore, the mRNA transcription levels of the SVCV-G gene after transfection with shViperins were detected by qRT-PCR. Compared to the control group, the SVCV copy numbers were significantly increased at 48 h and 72 h, being 12 and 15 times lower than those in the shNC group at 48 h and 72 h (Figure 7A). The expression of the SVCV-G protein after SVCV infection for 48 h and 72 h was significantly decreased (Figure 7B). These results indicate that the downregulation of viperin protein expression could promote SVCV replication.

## 4. Discussion

Teleost fish possess a complete immune system as a result of evolution, although they mainly depend on their innate immunity when dealing with pathogens [29]. The induction of type I interferons (IFN) is critical for the antiviral innate immune response, and then IFNs induce hundreds of interferon-stimulated genes (ISG) to exert antiviral effects. *Viperin*, as an important ISG, has important roles in antiviral immunity. In this research, we identified the *viperin* gene of common carp (*cc-viperin*) and investigated its effects on SVCV infection.

Viperin is a member of the radical S-adenosyl-l-methionine (SAM) superfamily. It contains three main domains: an N-terminal amphipathic α-helix domain, a radical SAM domain, and a highly conserved C-terminal domain [30]. There are sequence differences in the N-terminal of *viperin* among species, but the central domain and C-terminal display high conformation, so viperin exhibits higher similarity among some species [3]. In this study, the full sequence of the *cc-viperin* cDNA was 1044 bp long and encoded 348 amino acids. Its molecular weight was 40.4 kDa. A previous study has shown that the human *viperin* gene encodes 361 amino acids and the protein molecular weight is also 40.4 kDa. The sequence comparison and phylogenetic analysis show that the *cc-viperin* sequence is similar to those of other species, especially bony fish. This reveals a recent evolutionary relationship among *cc-viperin* and that of *crucian carp* and black carp. The radical SAM domain of *cc-viperin* is located between 60 aa and 268 aa, containing a three-cysteine motif, CxxxCxxC, which is similar to that of others. However, the 70-amino-acid region of the N-terminal is different from that of other species. The CxxxCxxC domain exhibits high homology among species and can bind with the catalytic [4Fe-4S] cluster via the SAM-dependent radical mechanism, which inhibits viral replication [30]. Therefore, in view of the differences and similarities in the characteristics of viperin sequences among species, can we speculate that the similar functions of viperin may be mainly related to its conserved sequences, while the differences may be related to regions with greater difference?

Viperin is usually localized in the endoplasmic reticulum (ER) or lipid droplets via its N-terminal amphipathic α-helix, but it also seems to be localized in other organelles at different physiological states. For example, viperin was localized to mitochondria in human foreskin fibroblasts during HCMV infection [31]. Grouper viperin was observed in the cytoplasm and co-localized in the ER [32]. *Crucian carp* viperin is a cytoplasmic protein associated with the ER [5]. Here, cc-viperin was also found to be localized in the cytoplasm. These findings show that fish viperins occupy the same locations in cells to perform their functions. On the other hand, the cellular localization of viperin is inconsistent in different animals, and more research is necessary to examine this.

Teleost fish play an important role in evolution because they are the earliest known vertebrates possessing a complete immune system. They mainly depend on their innate immunity when dealing with infections [29]. Viperin is increasingly being considered as a key protein contributing to the host’s defense mechanisms, highlighting its involvement in the immune response to viral threats by inhibiting the replication of various viruses [4]. The *viperin* gene is always expressed in normal cells at very low basal levels, but its upregulation can be induced in response to pathogens or corresponding analogs, such as viruses, bacteria, type I IFNs, lipopolysaccharide (LPS), and poly(I:C) in vitro [5,33]. In studies of aquatic diseases, viperin has been given widespread attention. For example, *viperin* expression was exhibited in response to the salmon anemia virus (ISAV) in Atlantic salmon (*Salmo salar*) [13], grass carp reovirus (GCRV) infection in crucian carp (*Carassius auratus*) [34], and in groupers against Singapore grouper iridovirus (SGIV) [32]. SVCV, as a serious deadly virus, can cause high mortality and significant economic losses in the carp breeding industry. In this study, we found that SVCV infection could increase the *viperin* gene’s expression levels in EPC cells. *cc-viperin* overexpression displayed inhibitory effects on SVCV replication, and the knockdown of *cc-viperin* gene expression via siRNA interference could promote the replication of SVCV. These findings suggest that SVCV infection can indeed induce *viperin* gene expression and that the viperins do inhibit SVCV replication. The results are similar to those of previous studies. They again confirm the antiviral function of viperin in animals. Previous research concluded that viperin exerts inhibitory effects on viral replication function via different functions: (1) viperin catalyzes the conversion of cytidine triphosphate (CTP) to 3′-deoxy-3′, 4′-didehydro-CTP (ddhCTP) by the SAM-dependent radical mechanism; (2) viperin interacts with host proteins involved in innate immune signaling during their lifecycle [35]. These processes essentially inhibit viral replication or the host cell’s immune response [36]. Although viperin may have the above mechanisms, its underlying antiviral mechanism in fish remains unclear. Here, the mechanisms by which viperin inhibited SVCV replication in EPC cells were based on the above two pathways; other potential pathways need further study in the future.

## 5. Conclusions

*Viperin*, as an important ISG, plays an important role in the antiviral immunity of vertebrates. In this paper, the identification, characterization, and antiviral effects of the common carp *viperin* gene were presented. *cc-viperin* showed similar gene characteristics to other species and displayed inhibitory effects on SVCV replication. This research is not only an extension of *viperin* gene research but also contributes to basic research on viral diseases in aquatic animals.

## Figures and Tables

**Table 1:** Primer sequences used in this study.

Primer	Sequence (5′-3′)	Size (bp)	Application
Viperin-F	ATGTTAATGCAATTTTGTTTC	1044	PCR
Viperin-R	TCACCACTCCAGTTTCATATC		
Viperin-F1	TACCGGACTCAGATCTCGAGATGTTAATGCAATTTTGTTTC	1044	
Viperin-R1	CGACTGCAGAATTCGAAGCTTCCACTCCAGTTTCATATC		
β-actin-F	GCCGTGACCTGACAGACTAC	122	qPCR
β-actin-R	GTCAAGAGCCACATAGCAGAG		
Viperin-qF	CGCACCAAAGAGCAGAAAGA	138	
Viperin-qR	AATGGGCAAGACGAAAGAGG		
qSVCV-F	CGACCTGGATTAGACTTG	182	
qSVCV-R	AATGTTCCGTTTCTCACT		
shRNA-179	GCAGTGTGAACTACCACTTTATTCAAGAGATAAAGTGGTAGTTCACACTGCTT		RNA interference
shRNA-756	GGCAGAGAAATTCGTTATTAGTTCAAGAGACTAATAACGAATTTCTCTGCCTT		
shRNA-1016	GCAGTGTGAACTACCACTTTATTCAAGAGATAAAGTGGTAGTTCACACTGCTT		
shRNA-NC	GTTCTCCGAACGTGTCACGTTTCAAGAGAACGTGACACGTTCGGAGAACTT		

## Data Availability

The data supporting the findings are available within the article.

## Supplementary Materials

The following supporting information can be downloaded at: https://www.mdpi.com/article/10.3390/ani15010096/s1. File S1 The original western blot images of Figure 4, Figure 5, Figure 6 and Figure 7.
