# Supplementary figures and images for: Viperin and Its Effect on SVCV Replication in Common Carp, Cyprinus carpio
Source: Animals (Basel). 2025 Jan 3;15(1):96. doi: 10.3390/ani15010096 (PMC11718829; doi:10.3390/ani15010096)

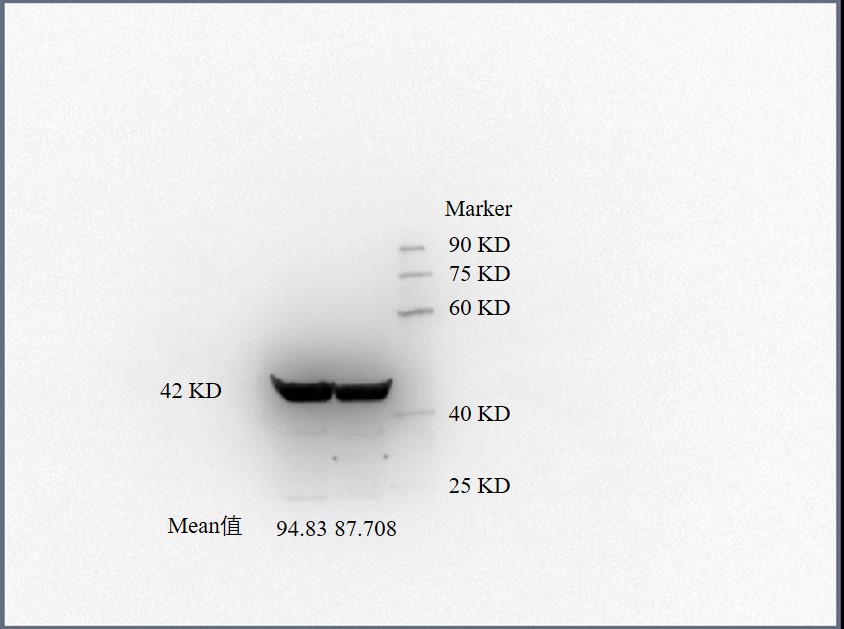

Supplement: Supplementary file 1 [file animals-15-00096-s001.zip › original western blot images WB20241216/Figure 4/figure 4 actin.jpg]

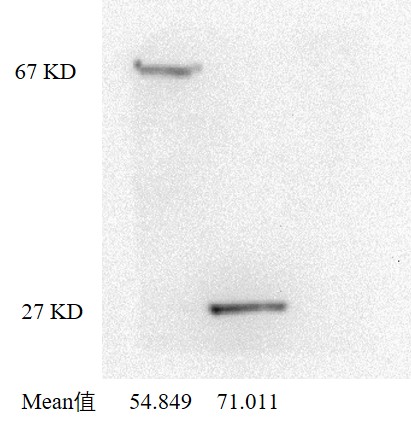

Supplement: Supplementary file 1 [file animals-15-00096-s001.zip › original western blot images WB20241216/Figure 4/figure 4.jpg]

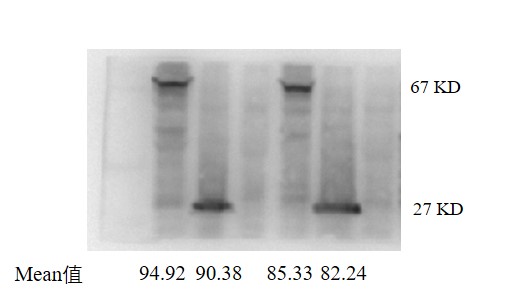

Supplement: Supplementary file 1 [file animals-15-00096-s001.zip › original western blot images WB20241216/Figure 5/figure 5A.jpg]

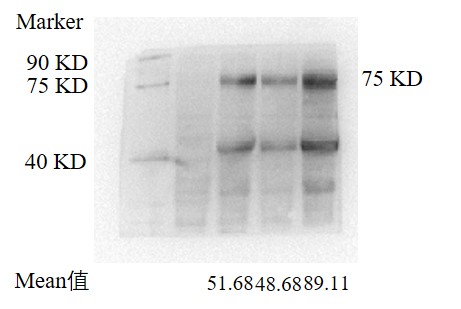

Supplement: Supplementary file 1 [file animals-15-00096-s001.zip › original western blot images WB20241216/Figure 5/figure 5C.jpg]

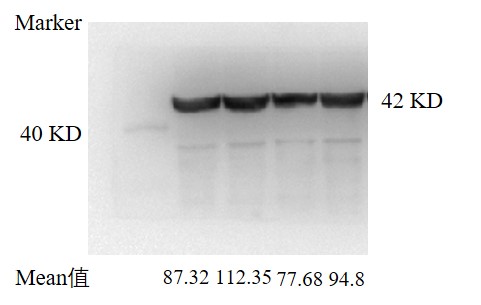

Supplement: Supplementary file 1 [file animals-15-00096-s001.zip › original western blot images WB20241216/Figure 5/figure5C actin.jpg]

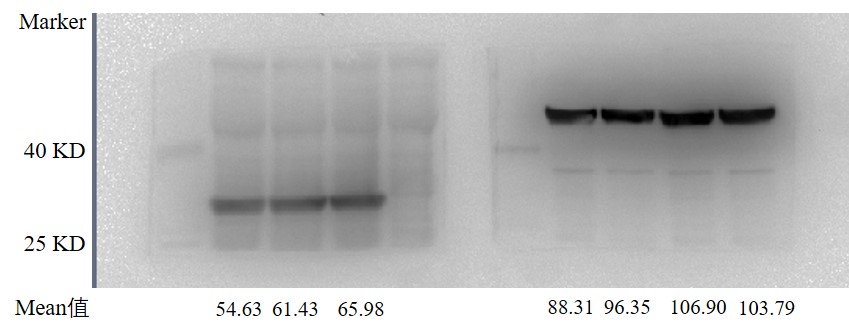

Supplement: Supplementary file 1 [file animals-15-00096-s001.zip › original western blot images WB20241216/Figure 6/figure 6B.jpg]

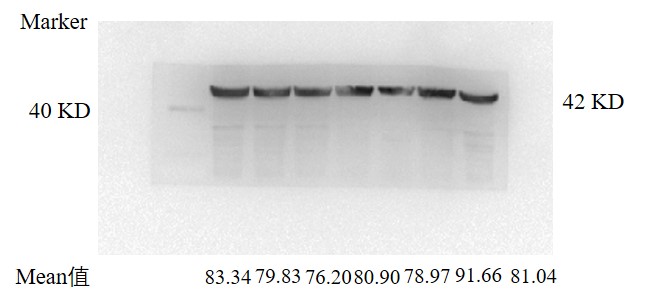

Supplement: Supplementary file 1 [file animals-15-00096-s001.zip › original western blot images WB20241216/Figure 7/figure 7B actin.jpg]

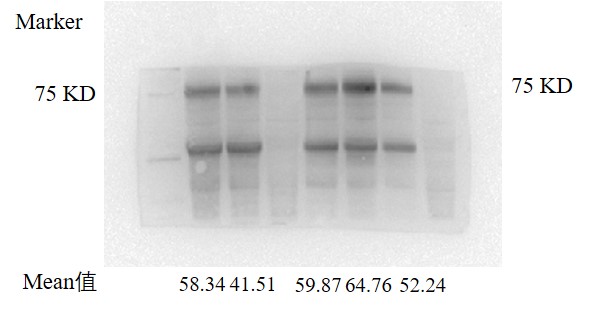

Supplement: Supplementary file 1 [file animals-15-00096-s001.zip › original western blot images WB20241216/Figure 7/figure 7B SVCV.jpg]
